# Effects of probiotic supplementation on immune and inflammatory markers in athletes: an umbrella review and re-analysis of published meta-analyses of randomised controlled trials

**DOI:** 10.7717/peerj.20809

**Published:** 2026-02-26

**Authors:** Lei Chen, Aichun Li, Wenhao Chen, Junlai Zhou, Yujia Kou

**Affiliations:** 1 School of Physical Education, Hainan Normal University, Haikou, China; 2 School of Sports Science and Health, East China Normal University, Shanghai, China

**Keywords:** Probiotics, Athletes, Immune function, Inflammatory factors, Umbrella review, Re-analysis

## Abstract

**Background:**

Intensive training and competition can compromise athletes’ immune function and elevate inflammatory responses. Although probiotics are widely studied as a nutritional intervention, existing meta-analyses have reported inconsistent findings regarding their efficacy. This umbrella review and re-analysis aimed to synthesize and evaluate the available evidence on the effects of probiotic supplementation on specific immune and inflammatory markers in athletes.

**Methods:**

We systematically searched PubMed, Cochrane Library, Web of Science, EMbase, and Scopus for meta-analyses published up to December 1, 2025, and supplemented these with recent randomised controlled trials (RCTs) (up to December 15, 2025). Quality was assessed using Assessment of Multiple Systematic Reviews 2 (AMSTAR2) and Grading of Recommendations, Assessment, Development, and Evaluation (GRADE) tools. Outcomes of interest included tumor necrosis factor-alpha (TNF-α), interleukin-6 (IL-6), interleukin-8 (IL-8), interleukin-10 (IL-10), immunoglobulin A (IgA) and interferon-gamma (IFN-γ). Overlap among studies was evaluated using the Graphical Representation of Overlap for OVErviews (GROOVE) tool, and data were re-analyzed using random- or fixed-effects models in Stata 15.0.

**Results:**

This umbrella analysis incorporated five meta-analyses (encompassing 69 RCTs) and one additional recent RCT, totaling 3,413 participants. Results showed that probiotic supplementation significantly reduced levels of the pro-inflammatory marker TNF-α (Effect Size (ES) = −0.59, 95% Confidence Interval (CI) [−0.94 to −0.24], *P* = 0.001). Probiotic supplementation significantly increased secretory IgA (ES = 0.30, 95% CI [0.03–0.57], *P* = 0.031) and IFN-γ levels (*P* < 0.01). In contrast, no significant effects were observed for IL-6 (ES = −0.09, 95% CI [−0.26 to 0.08]; *P* = 0.283), IL-8 (ES = −0.38, 95% CI [0.87 to 0.11], *P* = 0.132) and the anti-inflammatory cytokine IL-10 (ES = 0.15, 95% CI [−0.21 to 0.52], *P* = 0.411).

**Conclusions:**

These robust results demonstrate that probiotic supplementation modulates exercise-induced immune disturbances in athletes by attenuating pro-inflammatory responses and enhancing mucosal immunity. These findings support its role as a strategic nutritional approach for immune protection. Future research should emphasize strain-specific efficacy and optimal dosing to enable personalized recommendations for athletes.

## Introduction

Elite athletes endure cyclical immunometabolic remodeling during intensive training cycles (Kostiukevych et al., 2019). Exceeding immunotolerance thresholds triggers lymphocyte subset dysregulation and mucosal barrier impairment, consistent with exercise immunology’s “open window theory”—a 3–72 h post-exertion vulnerability period. Crucially, exercise demonstrates dose-dependent immunomodulation: moderate activity enhances Natural Killer (NK) cell surveillance and neutrophil phagocytosis, while sustained high-intensity training induces pathogenic cytokine overexpression with anti-inflammatory dysregulation, driving persistent immunoinflammatory imbalance (McFadden et al., 2023). This state elevates upper respiratory infection risk by 2–6-fold and disrupts neuroendocrine homeostasis, ultimately compromising athletic performance (Guimarães, Coelho & Rubini, 2022).

In recent years, probiotics, as potential immunomodulators and anti-inflammatory agents, have attracted widespread attention (Azad, Sarker & Wan, 2018). Probiotics are live microorganisms whose beneficial effects on host health, when ingested in appropriate amounts, have been confirmed in recent large-scale population trials (Sudaarsan & Ghosh, 2024; Tan, Navarro & Macia, 2023). Their mechanisms of action mainly include regulating the composition of intestinal flora, enhancing intestinal mucosal barrier function, and systematically regulating immune responses (Miniello et al., 2023). A number of clinical studies have also shown that specific probiotic strains can effectively enhance mucosal immunity, regulate cytokine expression, and reduce exercise-induced oxidative stress (Zhang, Zhang & Li, 2023; Patani et al., 2023). However, in randomised controlled trials (RCTs) conducted on athlete populations, there remains significant heterogeneity in the results (Mohr et al., 2024). In particular, disparate data exist regarding the effects of probiotic supplementation on key immune and inflammatory markers in athletes, such as salivary immunoglobulin A (sIgA), Interleukin-6 (IL-6), and C-reactive protein (CRP) (Fernández-Lázaro et al., 2023).

The reasons for the inconsistencies in existing research results are complex and diverse. First, the effects of probiotics are highly strain-specific (Rückle et al., 2022); different strains (*e.g.*, *Lactobacillus casei* Shirota and *Lactobacillus fermentum* PCC) or even different substrains within the same species may trigger distinct immune responses (Kurian et al., 2021). Second, there is a large variation in dosage ranges, with the common daily dosage ranging from 2 × 10^8^ to 1 × 10^1^^1^ colony-forming units (CFU), and effective dosage thresholds for different markers have not yet been established (Mohr et al., 2024). Third, intervention durations vary, and short-term studies often fail to capture adaptive changes in markers such as sIgA (Carretón, Morchón & Montoya-Alonso, 2017), while the responses of acute-phase proteins like CRP may also change over time (Pay & Shaw, 2019). Furthermore, there are significant individual differences among athletes, and factors such as training load, dietary structure, and genetic background can all affect the response to probiotics (Hughes & Holscher, 2021).

Although several meta-analyses have attempted to synthesize evidence from RCTs, their conclusions are hampered by methodological limitations, substantial heterogeneity, and inconsistent outcome measures (Fernández-Lázaro et al., 2023; Guo et al., 2022). While existing syntheses suggest that probiotic supplementation can reduce the severity of upper respiratory tract infections (URTIs) and lower pro-inflammatory cytokines like IL-6 and TNF-α in athletes (Łagowska & Bajerska, 2021; Maryam et al., 2020), and may even enhance performance in aerobic exercise (Asier et al., 2022), the optimal parameters for efficacy—such as strain type, dosage, and intervention duration—remain contentious (Li, Ruhao & Lu, 2023). Conventional meta-analyses are limited in their ability to reconcile these critical differences and often lack systematic evaluations of evidence strength and publication bias (Bungau et al., 2021; Zhou, 2015).

Therefore, to address the inconsistencies in the existing evidence and comprehensively evaluate its reliability, this study employed an umbrella review approach to systematically integrate and re-analyze data from existing published meta-analyses and newly available RCTs (Cuijpers et al., 2022). The core of this methodology involved the structured extraction and synthesis of evidence from multiple meta-analyses, coupled with standardized re-calculation based on original data, in order to overcome heterogeneity and conclusion conflicts arising from variations in study design, population characteristics, and outcome measures. By harmonizing effect sizes, assessing risk of bias, and verifying the robustness of the results, this study aims to objectively evaluate the efficacy and consistency of evidence regarding probiotics in alleviating exercise-induced inflammation and maintaining immune function, thereby providing a more comprehensive and integrated high-level evidence base for nutritional interventions in athletic populations.

### Methods

This study adhered to the Preferred Reporting Items for Systematic Reviews and meta-analyses (PRISMA) guidelines (Brian, David & Chris, 2015) and was prospectively registered with the International Prospective Register of Systematic Reviews (PROSPERO) under registration number CRD42024609963.

### Literature search strategy

A systematic literature search was performed independently by two researchers across five electronic databases: PubMed, Cochrane Library, Web of Science, EMbase, and Scopus. To comprehensively identify all relevant quantitative syntheses, the search strategy was constructed using core concepts from the PICOS framework, focusing on the population (athletes) and intervention (probiotics). The primary search for meta-analyses, conducted up to December 1, 2025, utilized the key terms “Athletes”, “Probiotics”, and “Meta-Analysis” without specifying outcomes, to maximize sensitivity given the limited number of meta-analyses in this specific field. Subsequently, an updated search for recent RCTs was carried out from the latest date covered by the identified meta-analyses until December 15, 2025, using the terms “Athletes”, “Probiotics”, and “Randomized Controlled Trial”. The complete search strategies for all databases are provided in Tables S1–S5.

### Literature screening and data extraction

Between November 10, 2024 and December 20, 2025, two independent reviewers (W.C. and Y.K.) screened the titles, abstracts, and full texts of all retrieved studies. Any discrepancies were resolved through consensus discussion or, if necessary, adjudication by a third reviewer (A.L.). Data extraction was completed by December 23, 2025, and included key study characteristics (first author, publication year, sample size, probiotic strain, dosage, and intervention duration). Outcome data, including effect sizes (ES) and corresponding 95% confidence intervals (CIs) for immune and inflammatory biomarkers, were systematically extracted.

### Inclusion and exclusion criteria

Study selection was rigorously guided by the pre-defined PICOS criteria detailed in Table 1. For the umbrella review, we included published systematic reviews that incorporated a meta-analysis of RCTs involving athletes supplementing with probiotics *versus* a placebo or no intervention. Although the initial search was broad, the screening process strictly enforced the eligibility criteria, specifically selecting studies that reported outcomes for pre-specified immune and inflammatory markers: TNF-α, IL-6, IL-8, IL-10, IgA, IFN-γ, and CRP. To incorporate the most recent evidence, RCTs published after the search date of the included meta-analyses were also eligible. We excluded non-randomized studies, preclinical research, observational designs, quasi-experimental trials, duplicate reports, and articles with unavailable full texts.

**Table 1:** PICOS for probiotic supplementation in athletes.

**PICOS element**	**Description**
Population	Participants included both amateur and professional athletes engaged in systematic training and organized competitions, excluding occasional participants or individuals without regular training. All selected individuals were in good health, with no chronic immunological disorders or acute infections.
Intervention	Probiotic supplementation in any form (e.g., capsules, powder, fortified food) regardless of strain, dosage, or duration. No minimum dosage threshold was applied; however, all strain types, dosages (CFU), and intervention periods were extracted and reported.
Comparison	Placebo or no intervention.
Outcomes	Changes in immune and inflammatory biomarkers, including: • Pro-inflammatory cytokines: TNF-α, IL-6, IL-8 • Anti-inflammatory cytokine: IL-10 • Immunoglobulin: salivary IgA • Interferon: IFN-γ • Acute-phase protein: CRP Other relevant immune markers were also considered if reported.
Study design	Meta-analyses of randomized controlled trials (RCTs) and additional RCTs published in English or Chinese. Studies must provide sufficient data for effect size calculation or qualitative synthesis.

### Quality assessment

The quality of meta-analyses was assessed using the Assessment of Multiple Systematic Reviews (AMSTAR2) and the Grading of Recommendations, Assessment, Development, and Evaluation (GRADE). Supplementary RCTs were assessed using the Cochrane Risk of Bias tool. AMSTAR2 consists of 16 items categorized as high, moderate, low, or very low quality (Nemet et al., 2020). GRADE assesses the quality of evidence in terms of five dimensions: limitations, inconsistencies, indirectness, imprecision, and publication bias (Guyatt et al., 2011). The Cochrane tool assesses random sequence generation of supplemental RCTs, allocation concealment, use of blinding, outcome completeness, selective reporting, and other biases (Gu, Wang & Li, 2014). All of the above evaluations were independently evaluated by 2 researchers (L.C. and J.Z.) and then cross-reviewed, with a third expert (A.L.) adjudicating in case of disagreement.

### Literature overlap

This study used the “Graphical Representation of Overlap for OVErviews (GROOVE)” tool (Perez-Bracchiglione et al., 2022) to assess the degree of literature overlap among outcome indicators, with corrected coverage area (CCA) values as the quantitative indicator. CCA values ≤ 5% were considered low overlap, allowing for direct analysis; while CCA values ≥ 6% indicated significant overlap and potential bias risk (Ma et al., 2024). To eliminate bias, highly overlapping literature sets were identified based on CCA. Priority was given to retaining studies with larger sample sizes or better methodological quality, removing duplicate studies, and constructing a non-overlapping literature set. Based on this set, data were re-extracted and pooled analyses were conducted. Additionally, sensitivity analysis was performed to enhance the robustness and transparency of the evidence in this study.

### Statistical analysis

All statistical analyses were performed using Stata 15.0. Pooled effect sizes with 95% CIs were calculated through meta-analysis, with fixed-effects models applied for low heterogeneity (Cochran’s Q test *P* ≥ 0.10 and *I*^2^ ≤ 50%) and random-effects models for significant heterogeneity. Results were visualized in forest plots, supported by sensitivity analyses to evaluate robustness. Publication bias was assessed using Egger’s regression test (*P* < 0.05 considered significant), with trim-and-fill analysis conducted to adjust for potential missing studies.

## Results

### Literature screening result

**Figure 1:**
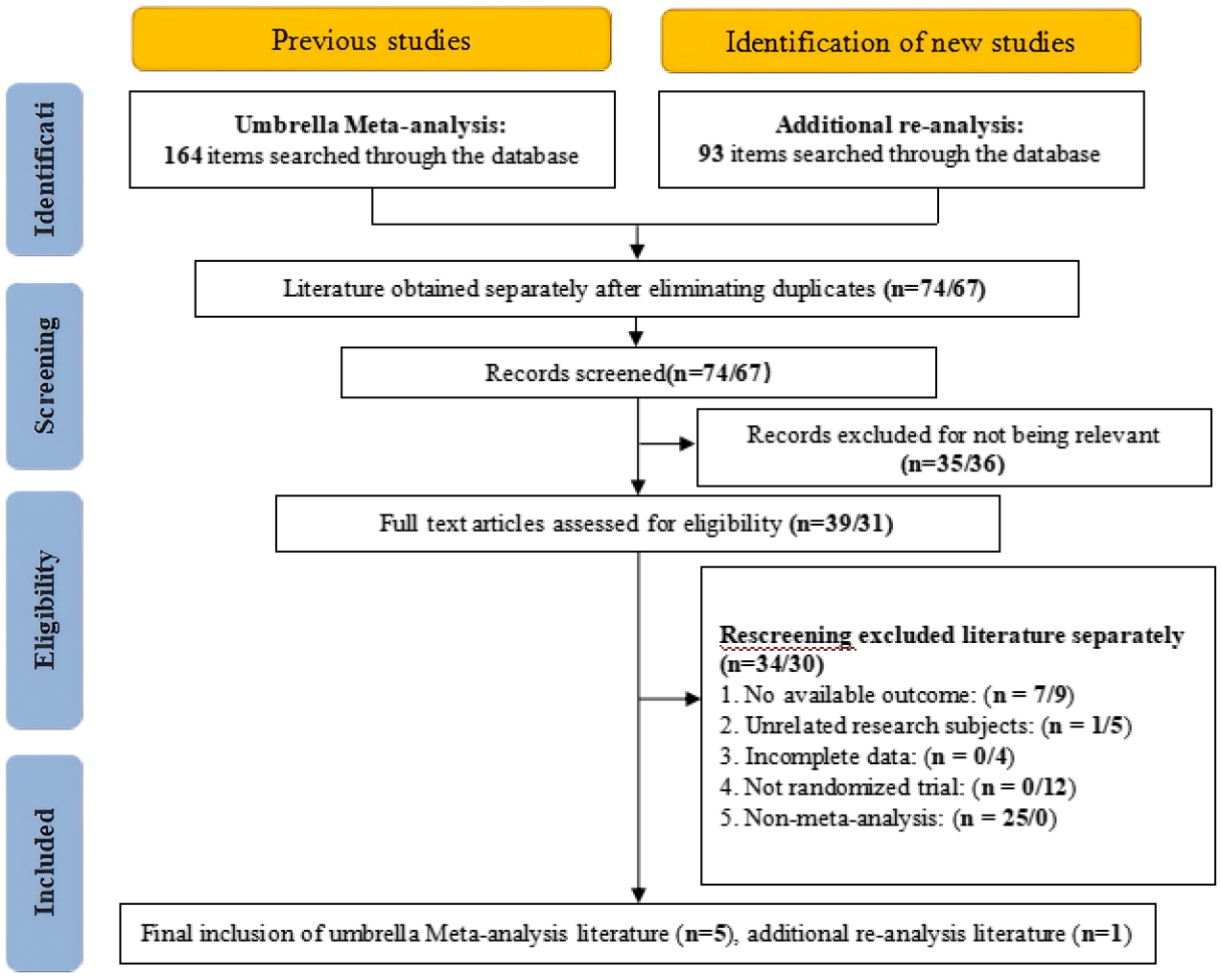
Umbrella meta-analysis and supplemental re-evaluation literature screening process.

Following a comprehensive search of five databases and subsequent screening of titles, abstracts, and full texts, five meta-analyses (Guo et al., 2022; Łagowska & Bajerska, 2021; Maryam et al., 2020; Tavakoly et al., 2021; Aparicio-Pascual et al., 2025) were ultimately included in this study. Furthermore, to provide the most up-to-date evidence, one RCT (Tavares-Silva et al., 2024) published between June 28, 2024, and December 15, 2025, was incorporated for supplementary analysis. The detailed literature selection process is illustrated in Fig. 1. The baseline characteristics of the included studies are presented in Table 2 and Table 3, while detailed characteristics of the athletes in the primary studies are provided in Table S6.

**Table 2:** Basic characteristics of the included studies.

**Author** **(Year)**	**Country**	**Literature search time frame**	**No. of studies**	**Duration time**	**Number of participants (n)** **INT/CON**	**Age range** **(years)**	**Interventions**	**Comparisons**	**Subject of intervention**	**Bias of risk assessment**	**Outcome**
Aparicio- Pascual (2025)	Spain	As of June 28, 2024	17	1–14 weeks	240/247	18–50	*Bifidobacterium animalis* subsp. *Lactis*, *Bifidobacterium bifidum*, *Bifidobacterium breve*, *Bifidobacterium infantis*, *Bifidobacterium lactis*, *Bifidobacterium longum*, *Lactobacillus acidophilus*, *Lactobacillus brevis*, *actobacillus bulgaricus*, *Lactobacillus casei*, *Lactobacillus fermentum*, *Lactobacillus fructivorans*, *Lactobacillus helveticus*, *Lactobacillus paracasei*, *Lactobacillus plantarum*, *Lactobacillus rhamnosus*, *Lactobacillus salivarius*, *Lacticaseibacillus casei*, *Lactococcus lactis*, *Pediococcus acidilactici*, *treptococcus thermophilus*	Placebo	Endurance athlete	McMaster Scale	TNF−α↔ IFN−γ↔ IL−6↔ IL−8↔ IL−10↑
Guo (2022)	Taiwan	As of May 12, 2022	9	8–90 days	170/165	21–39	*Lactobacillus fermentum*, *Bifidobacterium bifidum*, *Bifidobacterium lactis*, *Lactobacillus acidophilus*, *Lactobacillus lactis*, *Lactobacillus paracasei*, *Bifidobacterium animalis* subspecies *Lactobacillus*, *Lactobacillus brvis* W63, *Lactobacillus casei*, *Lactobacillus salivarius*, *Bacillus subtilis*, *Bifidobacterium longum* ES1, *Enterococcus faecalis* W54, *Bifidobacterium breve*, *Lactobacillus rhamnosus* GG.	Placebo	Healthy Athletes	Cochrane	TNF−α↓ IFN−γ↑ IgA↑ IL−6↔ IL−8↔ IL−10↔
Maryam (2020)	Iran	As of February 2021	14	2–14 weeks	393	18–38	*Streptococcus thermophilus*, *Bifidobacterium breve*, *Bacillus subtilis*, *Lactobacillus fermentum*, *Lactobacillus salivarius*, *Lactobacillus acidophilus*, *Bifidobacterium bifidum*, *Bifidobacterium animalis*, *Lactobacillus reuteri*, *Lactobacillus rhamnosus*, *Lactobacillus casei*, *Lactobacillus plantarum*, *Lactobacillus bifidus*, *Bacillus coagulans*, *Lactobacillus helveticus*, *Bifidobacterium infantis*, *Bifidobacterium longum*, *Lactococcus lactis*, *Lactobacillus shortum*, *Enterococcus faecalis*.	Placebo	Adult Athletes	Jadad	TNF−α↓ IFN−γ↑ IL−6↓ IL−8↔ IL−10↔
Karolina (2021)	Poland	February 1, 2020– September 30, 2020	14	1–17 weeks	771/538	19–40	*Streptococcus thermophilus*, *Bifidobacterium breve*, *Bacillus subtilis*, *Lactobacillus fermentum*, *Lactobacillus salivarius*, *Lactobacillus acidophilus*, *Bifidobacterium bifidum*, *Bifidobacterium animalis*, *Lactobacillus reuter*, *Lactobacillus rhamnosus*, *Lactobacillus casei*, *Lactobacillus plantarum*, *Bifidobacterium lactis*, *Bacillus coagulans*, *Bifidobacterium helveticus*, *Bifidobacterium infantis*, *Bifidobacterium longum*, *Lactobacillus acidophilus*, *Bifidobacterium brevis*, *Lactococcus faecium*, *Lactobacillus casei Saccharomyces boulardii*.	Placebo	Healthy adult professional athletes	Cochrane	TNF−α↓ IgA↔ IL−6↓ IL−10↑
Tavakoly (2021)	Iran	As of July 2020	13	2–20 weeks	836	20–40	*Bacillus subtilis*, *Lactobacillus casei*, *Lactobacillus acidophilus*, *Bifidobacterium bifidum*, *Bifidobacterium longum*, *Bifidobacterium infantis*, *Bifidobacterium animalis*, *Lactobacillus rhamnosus*, *Lactobacillus plantarum*, *Lactobacillus fermentum*, *Bifidobacterium lactis*, *Bifidobacterium breve*, *Streptococcus thermophilus*, *Enterococcus faecalis*, *Lactobacillus salivarius*, *Lactobacillus galli*.	Powder	Adult Athletes	Cochrane	IgA↔

**Notes.**

INT interventions CON comparations TNF tumor necrosis factor IFN Interferon IgA immunoglobulin A IL interleukin ↓ significantly decreased ↑ significantly increased ↔ no statistically significant difference

**Table 3:** Additional inclusion of essential features of reevaluation studies.

**Author** **(Year)**	**Country**	**Subject of intervention**	**Number of participants (n)** **INT/CON**	**Age range**	**Interventions**	**Comparison**	**Duration time**	**Outcome**	**Quality assessment** **(Cochrane)**
Tavares-Silva (2024)	Brazil	Male marathon runners	7 / 7	30–45 years	2.0 g/day probiotic capsules containing *Lactobacillus* *acidophilus*, *Lactobacillus* *lactis*, *Bifidobacterium* *lactis*, *Bifidobacterium* *bifidum* (1 × 10^9^ CFU per strain)	placebo (2.0 g cornstarch/day)	30 days	TNF-α, IL-2, IL-4, IL-6, IL-10,	⊖⨁⨁⨁⨁○ High risk

**Notes.**

TNF tumor necrosis factor IL interleukin ⨁ Low risk ⊖ Uncertain risk ○ High risk

### Characteristics of included studies and quality assessment results

**Figure 2:**
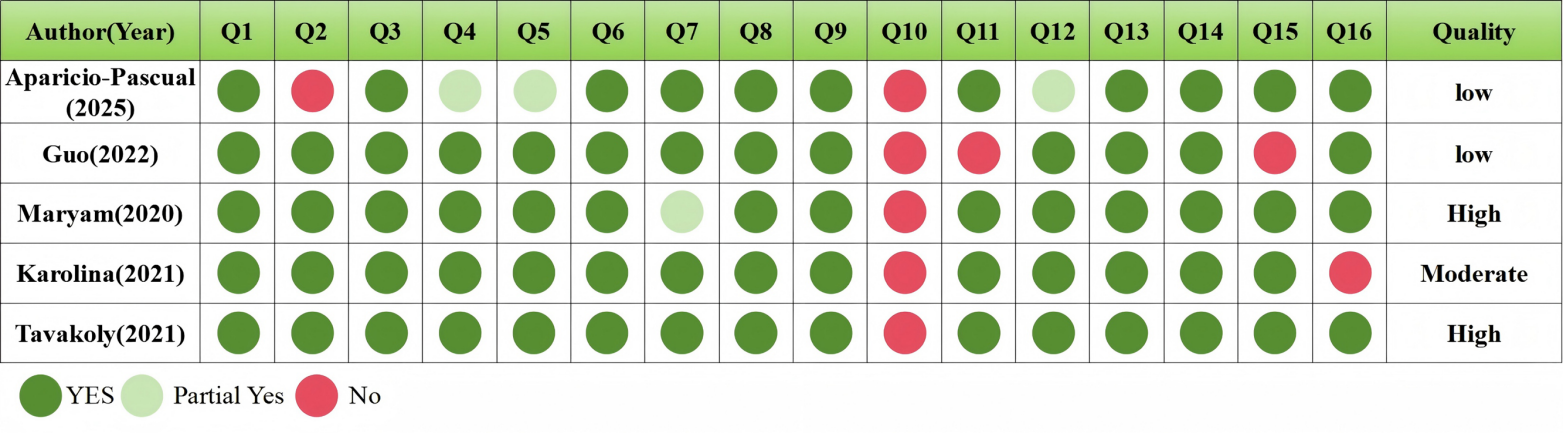
Results of AMSTAR2 quality assessment of literature included in meta-analysis. The 16 domains of the AMSTAR 2 checklist used for methodological quality assessment of systematic reviews, which include: (1) clarity of PICO components in research questions and inclusion criteria; (2) presence and adherence to a pre-established methodology; (3) transparency in specifying included study types; (4) comprehensiveness of the literature search strategy; (5) independent dual screening of studies to ensure selection consistency; (6) independent dual data extraction to enhance data reliability; (7) provision of a list of excluded studies with justifications; (8) detailed description of included study characteristics; (9) use of appropriate tools to assess risk of bias in included studies; (10) reporting of funding sources of included studies; (11) application of suitable statistical methods in meta-analysis; (12) assessment of the impact of risk of bias on meta-analysis results; (13) consideration of risk of bias during results interpretation; (14) discussion of heterogeneity in the findings; (15) investigation and discussion of publication bias where quantitative synthesis was performed; and (16) disclosure of all potential conflicts of interest, including systematic review funding sources.

This study included five meta-analyses (encompassing 69 original RCTs) and supplemented one additional RCT, with a total sample size of 3,413 participants. The methodological quality of the five meta-analyses was assessed using the AMSTAR 2 criteria, resulting in two being rated as high quality, one as moderate quality, and two as low quality, as detailed in Fig. 2. The supplementary RCT did not clearly report the blinding procedures and exhibited a small sample size with an all-male participant population, indicating potential selection bias. Consequently, it was categorized as high-risk of bias, as outlined in Table 3.

### Effect of probiotic supplementation on TNF-α in athletes

**Figure 3:**
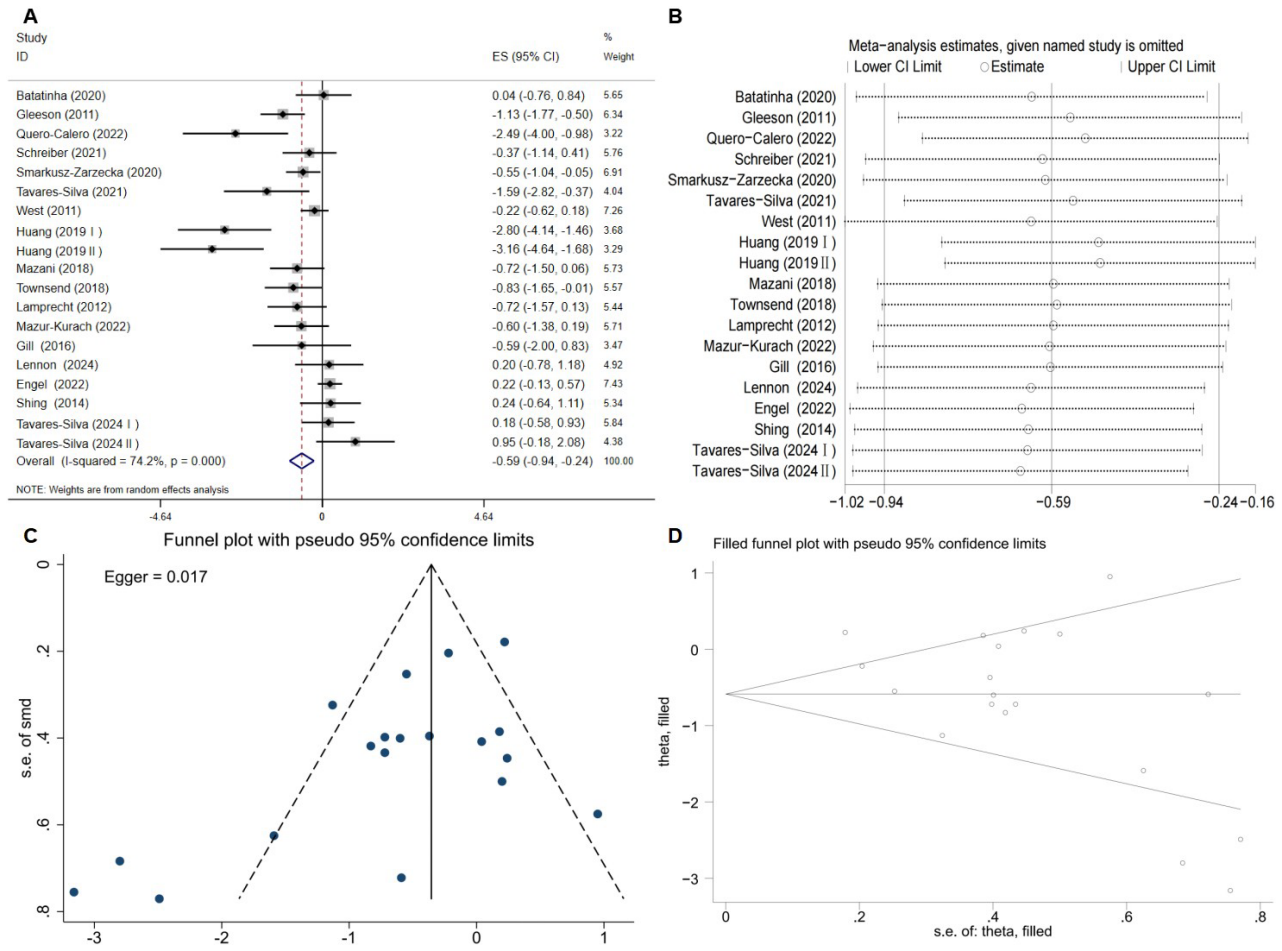
Evaluation of the effect of probiotic supplementation on TNF-α levels in athletes. (A) Forest plot from the random-effects meta-analysis. (B) Leave-one-out sensitivity analysis assessing the influence of individual studies. (C) Funnel plot for the visual assessment of potential publication bias. (D) Funnel plot after applying the trim-and-fill analysis to adjust for potential asymmetry.

An umbrella meta-analysis of four meta-analyses (Guo et al., 2022; Łagowska & Bajerska, 2021; Maryam et al., 2020; Aparicio-Pascual et al., 2025) (pooling MD and SMD effect sizes) indicated that probiotic supplementation significantly reduced TNF-α levels in athletes (*P* < 0.05; Table 4). Due to high overlap among the primary studies (CCA = 18.52%; Table 4), a re-analysis was performed after removing duplicate literature and incorporating one recent RCT (Tavares-Silva et al., 2024). The re-analysis results supported the above conclusion, showing that probiotics significantly lowered TNF-α levels (ES = −0.59, 95% CI [−0.94 to −0.24], *P* = 0.001; Fig. 3A). Although the analysis showed high heterogeneity (*I*^2^ = 74.2%, *P* < 0.001), leave-one-out sensitivity analysis demonstrated that the results remained stable (Fig. 3B). The funnel plot (Fig. 3C) and Egger’s test suggested potential publication bias (*P* = 0.017). However, the trim-and-fill analysis imputed no missing studies (Fig. 3D), suggesting the current results are robust.

**Table 4:** Results of umbrella meta-analysis.

**Author**, **Year**	**No. of studies**	**MA metric** **(MD, SMD, ES)**	**Heterogeneity**		**Effects model**	**MA outcomes**		**GRADE** **level**	**CCA**
			***I*** ^**2**^	***p*-value**		**ES** **(95% CI)**	***p*-value**		
**TNF-**α									
Aparicio- Pascual, 2025	15	SMD	80.57%	<0.001	Random	−0.28 (−0.73, 0.17)	0.218	○○○⨁ Very low	
Maryam, 2020	5	SMD	0%	0.96	Fixed	−0.72 (−1.11, −0.33)	<0.001	⨁○⨁⨁ Very low	
Guo, 2022	6	MD	70%	0.06	Random	−0.29 (−0.42, −0.16)	<0.01	○○⨁○ Very low	18.52% High overlap
Karolina, 2021	3	MD	91.92%	NA	Random	−2.31 (−4.12, −0.51)	0.01	○○⨁○ Very low	
ALL	2	ES SMD	52.3%	0.148	Random	−0.52 (−0.95, −0.09)	0.019	⨁○⨁⨁ Moderate	
	2	ES MD	79.10%	0.029	Random	−0.3 (−0.43, −0.17)	<0.001	⨁○⨁⨁ Moderate	
**IgA**									
Karolina, 2021	4	SMD	94.57%	NA	Random	0.24 (−0.09, 0.57)	0.44	○⨁⨁○ Low	
Guo, 2022	2	MD	0%	0.59	Fixed	3.57 (0.66, 6.48)	0.02	⨁⨁⨁○ Moderate	12.5% High overlap
Tavakoly, 2021	3	MD	0%	0.356	Fixed	−0.6 (−13.15, 11.95)	0.93	⨁⨁⨁⨁ High	
ALL	2	ES MD	0%	0.526	Fixed	3.36 (0.52, 6.19)	0.02	⨁⨁⨁⨁ High	
**IFN−γ**									
Guo, 2022	2	MD	97%	<0.001	Random	14.33 (13.76, 14.89)	<0.001	⨁○⨁○ Low	
Aparicio- Pascual, 2025	7	SMD	97.65%	<0.0001	Random	0.97 (−1.08, 3.02)	0.352	○○○⨁ Very low	0% Slight overlap
Maryam, 2020	4	SMD	86.15%	<0.001	Random	0.43 (0.09, 0.76)	0.012	⨁⨁○⨁ Very low	
ALL	2	ES SMD	0%	0.61	Random	0.44 (0.11, 0.78)	0.008	⨁⨁⨁⨁ High	
**IL−6**									
Aparicio- Pascual, 2025	18	SMD	25.11%	0.077	Random	−0.12 (−0.33, 0.08)	0.24	○⨁⨁○ Low	
Maryam, 2020	9	SMD	6.6%	0.38	Random	−0.58 (−0.87, −0.28)	<0.001	○⨁⨁⨁ Moderate	
Guo, 2022	5	MD	0%	0.41	Fixed	0.19 (−0.25, 0.63)	0.39	○⨁⨁○ Low	22.22% High overlap
Karolina, 2021	5	MD	81.99%	NA	Random	−2.52 (−4.39, −0.66)	0.002	○○⨁○ Very low	
ALL	2	ES SMD	84.1%	0.012	Random	−0.34 (−0.79, 0.11)	0.142	⨁○○⨁ Low	
	2	ES MD	87%	0.006	Random	−1.01 (−3.65, 1.63)	0.454	⨁○○⨁ Low	
**IL−8**									
Guo, 2022	4	MD	76%	0.005	Random	−0.57 (−1.33, 0.19)	0.14	○○⨁○ Very low	
Aparicio- Pascual, 2025	12	SMD	58.98%	0.011	Random	−0.16 (−0.50, 0.19)	0.376	○○⨁○ Very low	22.73% Very high overlap
Maryam, 2020	3	SMD	13.80%	0.313	Fixed	−0.15 (−0.68, 0.39)	0.594	○⨁○⨁ Low	
ALL	2	ES SMD	0%	0.975	Random	−0.16 (−0.45, 0.13)	0.288	⨁⨁⨁⨁ High	
**IL−10**									
Aparicio- Pascual, 2025	12	SMD	0%	0.791	Random	0.43 (0.25, 0.62)	<0.0001	○⨁⨁⨁ Moderate	
Maryam, 2020	6	SMD	0%	0.957	Fixed	−0.05 (−0.34, 0.24)	0.73	○⨁○⨁ Low	
Guo, 2022	5	MD	20%	0.29	Fixed	−0.1 (−0.19, −0.06)	0.0001	○⨁⨁⨁ Moderate	20% Very high overlap
Karolina, 2021	4	MD	94.57%	NA	Random	2.08 (0.37, 4.52)	0.1	○○⨁○ Very low	
ALL	2	ES SMD	86.6%	0.006	Random	0.20 (−0.27, 0.67)	0.396	⨁○○⨁ Low	
	2	ES MD	76.40%	0.04	Random	0.73 (−1.34, 2.81)	0.003	⨁○⨁⨁ Moderate	

**Notes.**

MA meta-analysis MD Mean difference SMD Standardized mean difference NA Not applicable GRADE Grading of Recommendations Assessment Development, and Evaluation CCA Common coverage area ⨁ High quality ○ Low quality (GRADE)

### Effect of probiotic supplementation on IgA in athletes

**Figure 4:**
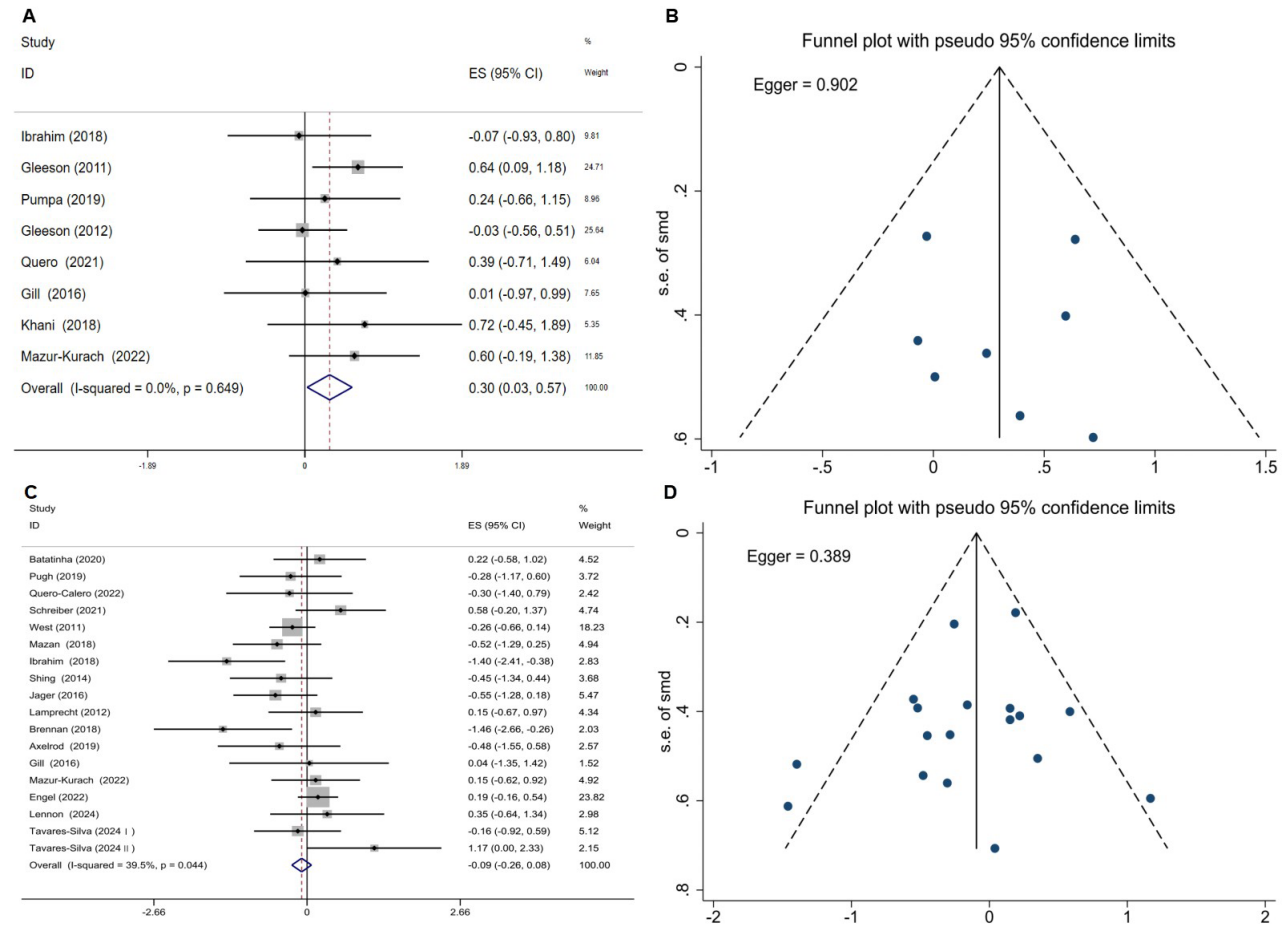
Meta-analytic evaluation of probiotic supplementation on IgA and IL-6 levels in athletes. (A) Forest plot from the fixed-effects meta-analysis of the effect on secretory IgA levels. (B) Funnel plot corresponding to the IgA meta-analysis for assessing publication bias. (C) Forest plot from the random-effects meta-analysis of the effect on IL-6 levels. (D) Funnel plot corresponding to the IL-6 meta-analysis for assessing publication bias.

Based on an umbrella review of existing meta-analyses, the analysis by Łagowska & Bajerska (2021) focusing on professional athletes in endurance disciplines (*e.g.*, running, cycling, and team sports) showed that probiotics had no significant effect on IgA levels (SMD = 0.24, 95% CI [−0.09 to 0.57], *P* = 0.44; Table 4). In contrast, two other pooled analyses (Guo et al., 2022; Tavakoly et al., 2021) involving mixed athlete populations (including endurance and team sports athletes) reported inconsistent findings (ES = 3.36, 95% CI [0.52–6.19]; *P* = 0.02). Further investigation revealed a high degree of overlap among the primary RCTs included in these analyses (CCA = 12.5%; Table 4). To address this, a re-analysis was performed after removing duplicate literature and incorporating one previously unincluded RCT (Mazur-Kurach, Barbara & Andrzej, 2022). The results indicated that probiotic supplementation significantly increased IgA levels in athletes (ES = 0.30, 95% CI [0.03–0.57], *P* = 0.031), with no observed heterogeneity among studies (*I*^2^ = 0%, *P* = 0.649; Fig. 4A). Neither the funnel plot nor Egger’s test suggested significant publication bias (*P* = 0.902; Fig. 4B).

### Effect of probiotic supplementation on IFN-γ in athletes

A meta-analysis (Guo et al., 2022) (MD = 14.33, 95% CI [13.76–14.89]; *P* < 0.001) and pooled analysis of two relevant studies (Maryam et al., 2020; Aparicio-Pascual et al., 2025) (SMD = 0.44, 95% CI [0.11–0.78]; *P* = 0.008) demonstrated that probiotic supplementation significantly elevates IFN-γ levels in athletes (Table 4). No overlap existed among the included original studies (CCA = 0%; Table 4), indicating the reliability of the existing research findings.

### Effect of probiotic supplementation on IL-6 in athletes

An umbrella meta-analysis of four meta-analyses (Guo et al., 2022; Łagowska & Bajerska, 2021; Maryam et al., 2020; Tavakoly et al., 2021) (pooling MD and SMD effect sizes) indicated that probiotic supplementation did not significantly affect IL-6 levels in athletes (*P* > 0.05; Table 4). Due to high overlap among the primary studies (CCA = 22.22%; Table 4), a re-analysis was performed after removing duplicate literature and incorporating one recent RCT (Tavares-Silva et al., 2024). The reanalysis results were consistent with the previous findings (SMD = −0.09, 95% CI [−0.26 to 0.08]; *P* = 0.283), further indicating that probiotic supplementation has no significant effect on IL-6 levels in athletes. Low heterogeneity was observed (*I*^2^ = 39.5%, *P* = 0.044), as shown in Fig. 4C. The Egger test revealed no significant publication bias (*P* = 0.389; Fig. 4D).

### Effect of probiotic supplementation on IL-8 in athletes

**Figure 5:**
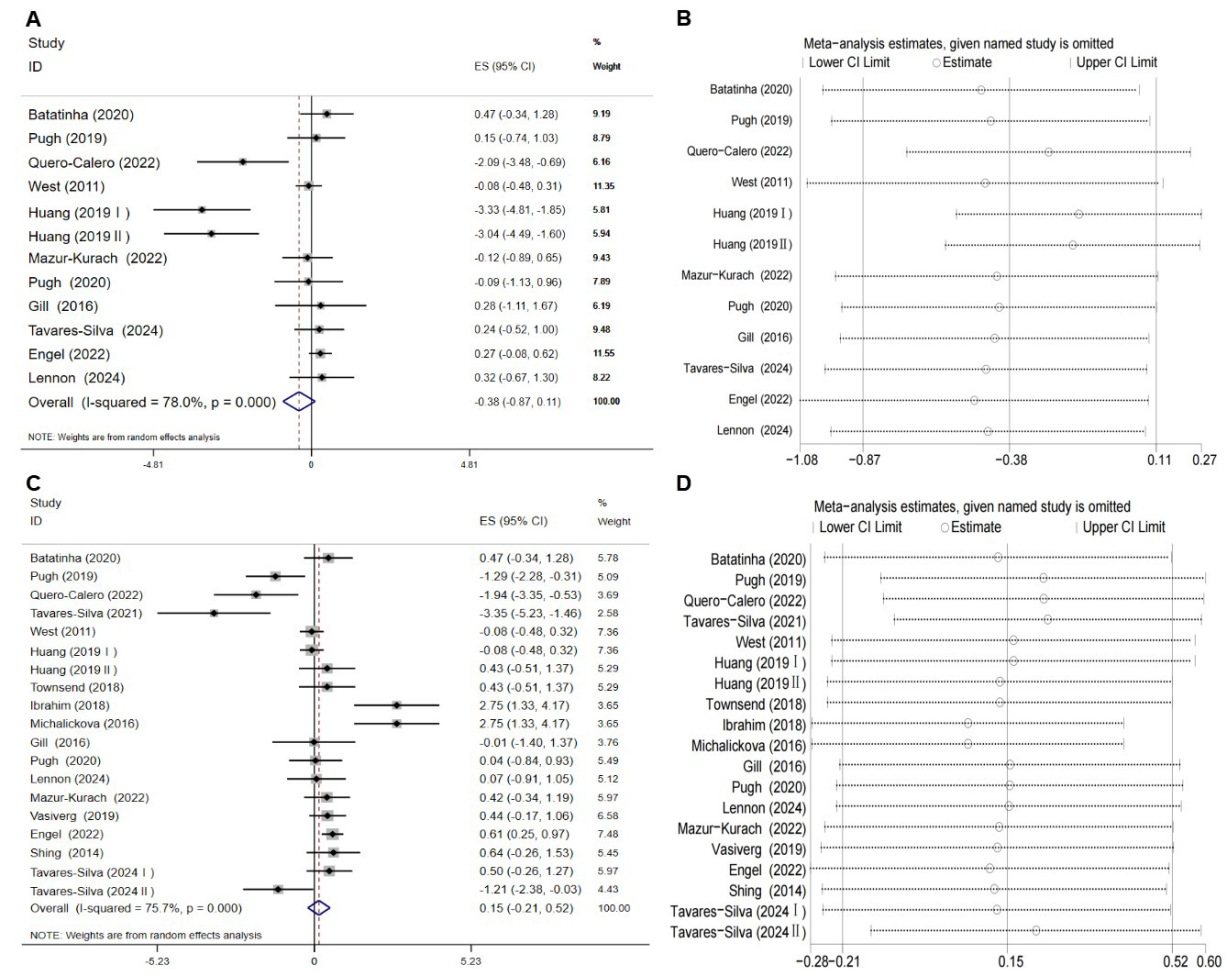
Meta-analysis of probiotic supplementation on IL-8 and IL-10 levels in athletes. (A) Forest plot from the random-effects meta-analysis of the effect on IL-8 levels. (B) Leave-one-out sensitivity analysis corresponding to the IL-8 meta-analysis. (C) Forest plot from the random-effects meta-analysis of the effect on IL-10 levels. (D) Leave-one-out sensitivity analysis corresponding to the IL-10 meta-analysis.

**Figure 6:**
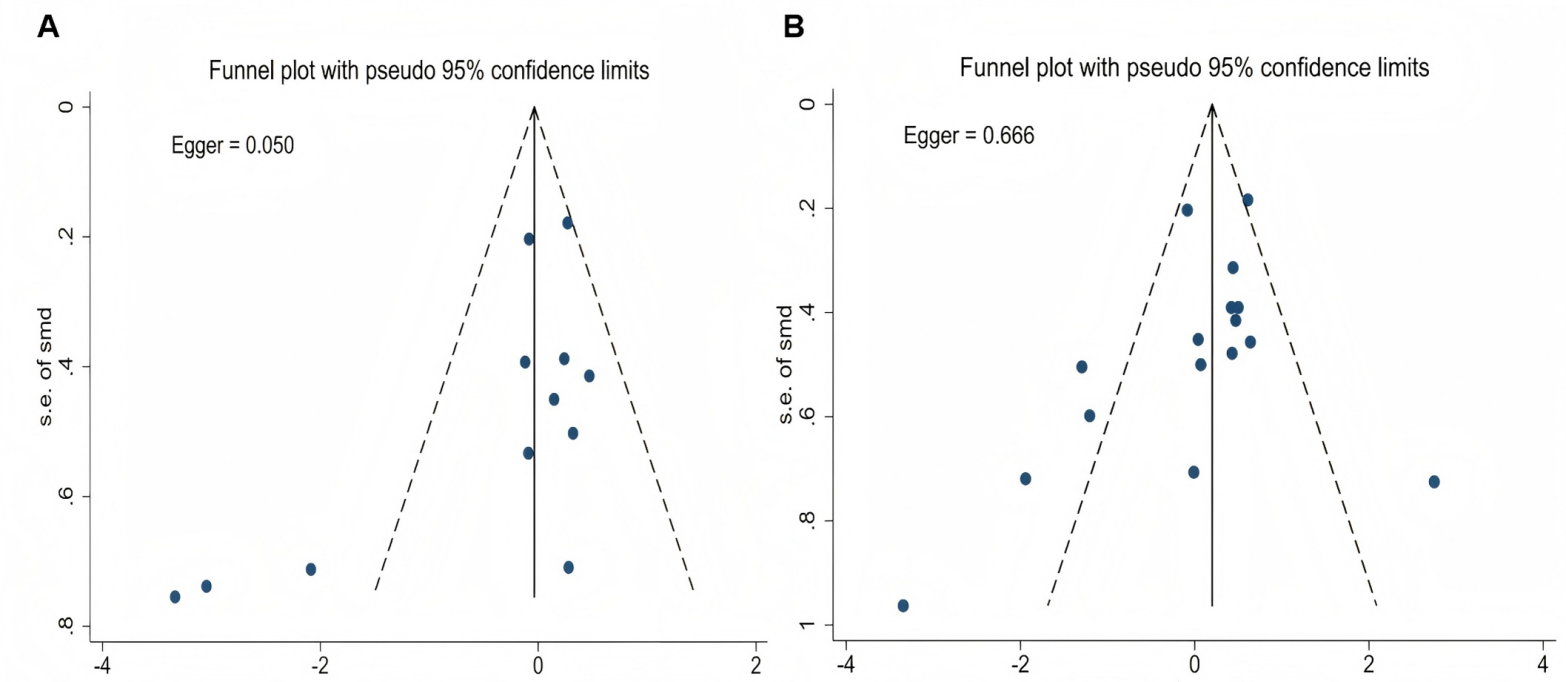
Assessment of publication bias for IL-8 and IL-10 meta-analyses. (A) Funnel plot for the meta-analysis evaluating the effect of probiotic supplementation on IL-8 levels. (B) Funnel plot for the meta-analysis evaluating the effect of probiotic supplementation on IL-10 levels.

An umbrella review of three related meta-analyses (Guo et al., 2022; Maryam et al., 2020; Aparicio-Pascual et al., 2025) indicated that probiotic supplementation had no significant effect on IL-8 levels in athletes (Table 4). Due to the high overlap among the primary studies included in these analyses (CCA = 22.73%), a re-analysis was conducted after removing duplicate literature. The results confirmed that probiotic supplementation continued to show no significant effect on IL-8 levels (SMD = −0.38, 95% CI [−0.87 to 0.11]; *P* = 0.132; Fig. 5A). Although high heterogeneity was observed (*I*^2^ = 78%, *P* < 0.001), sensitivity analysis demonstrated that the pooled effect size was not significantly influenced by any single study (Fig. 5B). Egger’s test did not detect significant publication bias (*P* = 0.05; Fig. 6A).

### Effect of probiotic supplementation on IL-10 in athletes

An umbrella meta-analysis of four meta-analyses (Guo et al., 2022; Łagowska & Bajerska, 2021; Maryam et al., 2020; Tavakoly et al., 2021) (pooling MD and SMD effect sizes) indicated that probiotic supplementation did not significantly affect IL-10 levels in athletes (*P* > 0.05; Table 4). Due to high overlap among the included primary studies (CCA = 16.67%; Table 4), a re-analysis was performed after removing duplicate literature and incorporating one recent RCT (Tavares-Silva et al., 2024). The re-analysis results were consistent with previous findings (SMD = 0.15, 95% CI [−0.21 to 0.52]; *P* = 0.411), further indicating that probiotics have no significant effect on IL-10 levels (Fig. 5C). High heterogeneity was observed in the results (*I*^2^ = 75.7%, *P* < 0.001), but sensitivity analysis suggested that the pooled effect size was relatively stable (Fig. 5D). Egger’s test did not detect significant publication bias (*P* = 0.666; Fig. 6B).

## Discussion

To date, numerous studies have examined the effects of probiotic supplementation on markers related to immune and inflammatory function in athletes, but findings have been inconsistent. Therefore, in order to obtain more definitive conclusions, we conducted this umbrella review aimed at evaluating the effects of probiotic supplementation on immune responses and inflammatory factors in athletes. To the best of our knowledge, this study is the first umbrella meta-analysis to summarize the effects of probiotics on immune and inflammatory factor markers in athletes, covering the available evidence and implications regarding changes in immune and inflammatory factor markers with probiotic supplementation.

In this article, five meta-analyses of RCTs involving athletes were identified through a systematic search of five databases. A comprehensive evaluation revealed that three of these studies (Guo et al., 2022; Łagowska & Bajerska, 2021; Maryam et al., 2020) reported a significant reduction in TNF-α levels following probiotic supplementation, whereas Aparicio-Pascual et al. (2025) observed no significant effect. Additionally, results from two of the analyses (Guo et al., 2022; Maryam et al., 2020) indicated that probiotics significantly increased IgA levels. However, findings regarding the impact on IFN-γ, IL-6, IL-8, and IL-10 levels were inconsistent across studies. To synthesize these conflicting findings and critically evaluate the quality of existing evidence, an umbrella meta-analysis was conducted. The CCA method was employed to quantify overlap among primary studies, and one recently published RCT was incorporated to update the data. The pooled results demonstrated that probiotic supplementation significantly reduced TNF-α levels and increased IgA and IFN-γ concentrations in athletes, while no significant effects were observed for IL-6, IL-8, or IL-10.

The observed inconsistencies in the literature regarding cytokines such as TNF-α, IFN-γ, IL-6, IL-8, and IL-10 likely stem from considerable heterogeneity across studies, particularly regarding athlete populations, probiotic strains, dosing regimens, and intervention durations. As a key pro-inflammatory cytokine, TNF-α is commonly elevated in response to exercise-induced muscle damage and oxidative stress. These elevated levels may adversely affect athletic performance and health by inhibiting protein synthesis, delaying recovery, and impairing immune function (Vijayaraghava & Doreswamy, 2017). The present meta-analysis demonstrated that probiotic supplementation significantly reduced peripheral blood TNF-α concentrations in athletes, suggesting its potential to mitigate exercise-induced inflammation. This finding is consistent with results from several RCTs involving athletes. For example, Townsend et al. (2018) reported that 12 weeks of supplementation with *Bacillus subtilis* DE111 significantly decreased TNF-α levels in collegiate baseball players. Similarly, Trushina et al. (2024) found that a 23-day daily regimen of multi-strain probiotics (≥1.25 × 10^10^; CFU) combined with 40g of dietary fiber effectively suppressed post-exercise elevations in TNF-α levels in basketball players. Mechanistically, probiotics may downregulate the expression of pro-inflammatory factors such as TNF-α through synergistic actions involving the activation of intestinal epithelial cells and macrophages, modulation of key inflammatory signaling pathways (*e.g.*, NF-κB and MAPK), and enhancement of phagocytic activity in intestinal immune cells (Airena et al., 2020; Zhang et al., 2023). Furthermore, a systematic review by Marttinen et al. (2020) indicated that sustained supplementation with specific strains (*e.g.*, *Lactobacillus casei*, 4 × 10^10^ CFU/day) can positively modulate post-exercise TNF-α levels and protein oxidation markers in endurance athletes. Therefore, targeted supplementation with appropriately dosed probiotics may help optimize training adaptation and recovery processes in athletic populations.

As a pleiotropic cytokine produced by activated T lymphocytes and NK cells, IFN-γ critically regulates macrophage activation and antigen presentation (Xu et al., 2021). This cytokine exerts direct antimicrobial and antitumor effects through upregulation of antigen processing pathways and induction of major histocompatibility complex class II (MHC-II) expression (Wijdeven et al., 2018). IgA, the predominant antibody isotype in mucosal secretions, provides primary defense against pathogen invasion by preventing microbial adhesion to epithelial surfaces and neutralizing both intracellular and extracellular pathogens through toxin neutralization (Shakya et al., 2016). These key immunological components (IFN-γ and IgA) constitute fundamental elements of athletes’ immune defense systems. Probiotic interventions enhance host immunity through microbiota-dependent immunoregulatory mechanisms that increase gut microbial diversity, activate T-cell responses, and promote Th1 cell differentiation, ultimately elevating IFN-γ levels to bolster innate and adaptive antimicrobial defenses (Anjum et al., 2023; Komai-Koma et al., 2016). During sustained intensive exercise, secretory IgA emerges as the dominant immunoglobulin in gastrointestinal and respiratory mucosa (Wang et al., 2023), serving as an essential immunological barrier against pathogen penetration (Abokor et al., 2021). This mucosal protection becomes particularly crucial during prolonged exercise training when immune surveillance is compromised (Matthew et al., 2022). Probiotics augment athletes’ mucosal immunity through dual mechanisms that induce B-cell maturation to boost IgA secretion while enhancing IFN-γ production to potentiate cell-mediated immunity (Ferro et al., 2021). These synergistic immunomodulatory effects counteract exercise-induced immunosuppression and inflammation while accelerating post-exercise recovery and improving systemic physiological adaptation to training demands.

Our meta-analysis found that probiotic supplementation had no significant overall effect on IL-6, IL-8, or IL-10 levels in athletes. This lack of a significant effect, despite positive findings in some individual studies (Łagowska & Bajerska, 2021; Aparicio-Pascual et al., 2025; Karlic, Krammer & Haslberger, 2022; Batatinha et al., 2020), is likely attributable to the opposing influences of heterogeneous factors across studies, such as differences in probiotic strains, intervention duration, and athlete training status (Diego et al., 2023). Sensitivity analysis confirmed the robustness of these results, and Egger’s test indicated no significant publication bias, supporting the credibility of the findings. This outcome aligns with several studies in athletic populations but contrasts with some reports in non-athletes. This discrepancy may be explained by the strain-specific nature of probiotic mechanisms, whereby different strains exert distinct immunomodulatory effects (Adejumo et al., 2023). The screening of strains for specific immunomodulatory effects requires further in-depth study (Shin et al., 2019). Furthermore, the immunomodulatory effects of probiotics are modulated by the host’s physiological status. Complex factors—including diet, gut microbiota diversity, psychological stress, sleep, and overall health—can interfere with probiotic colonization, metabolism, or action pathways, thereby altering their impact on inflammatory cytokine levels (Cristofori et al., 2021). Additionally, Noorifard et al. (2020) reported that significant effects may only become apparent after prolonged supplementation (*e.g.*, >12 weeks), suggesting that duration is a critical variable. While probiotics and their metabolites modulate immune function *via* interactions with innate and adaptive pathways—for example, by influencing macrophage polarization and mucosal barrier integrity—these mechanisms do not necessarily result in consistent, measurable changes in systemic IL-6, IL-8, or IL-10 concentrations (Nemati, Ebrahimi & Montazeri-Najafabady, 2024). Thus, despite a sound theoretical basis for probiotic modulation of these cytokines, substantial inter-individual variability likely obscures any significant population-level effect.

This study has several limitations that should be considered when interpreting the findings. Firstly, significant statistical heterogeneity (*I*^2^ > 75%) was observed specifically for IL-8 and IL-10. This substantial variability limits the certainty of conclusions for these markers and underscores their exploratory nature, warranting cautious interpretation of these particular effect sizes. It is noteworthy that moderate-intensity activity may enhance immunity, whereas high-intensity training may suppress it (Felice et al., 2024); however, the included studies did not systematically report training intensity or volume, which restricted our ability to stratify the results based on exercise dosage. Additionally, the potential influence of diet quality, including habitual probiotic intake from food sources, was not consistently controlled or reported across the included studies, which may contribute to the heterogeneity in responses.

The included studies encompassed a heterogeneous mix of athlete types, probiotic strains, and dosing regimens. Such variability may contribute to the inconsistent findings observed across certain inflammatory markers. Unfortunately, the insufficient reporting of primary data prevented us from examining how training intensity or other factors might modify probiotic efficacy. Consequently, the generalizability of our findings to specific athletic populations or probiotic protocols may be limited. Despite these limitations, the application of random-effects models and robust sensitivity analyses strengthens our confidence in the direction of the overall effects. Furthermore, the comprehensive discussion of potential mechanisms and clinical consistency across studies reinforces the plausibility and biological credibility of the primary findings. Future research should prioritize well-designed RCTs that stratify analysis based on these critical factors, including detailed training load monitoring and assessment of actual athletic performance, while also accounting for the influence of genetic background, sleep patterns, and other lifestyle factors, to establish more personalized recommendations and determine whether immunomodulatory changes translate into tangible performance outcomes.

## Conclusions

This study assessed the effects of probiotic supplementation on immune function and inflammatory factor levels in athletes through an umbrella meta-analysis with supplementary re-analysis. The results indicated that based on 5 meta-analyses (encompassing 69 RCTs) and 1 additional RCT (total 3,413 participants), probiotic supplementation significantly decreased TNF-α levels, whereas no significant effects were observed on IL-6, IL-8, or IL-10; meanwhile, it significantly increased IFN-γ and IgA levels. These findings suggest that probiotics may enhance immune responses and mitigate exercise-induced inflammation in athletes. However, due to the substantial heterogeneity among the included studies, these results should be interpreted with caution. Future research should investigate the strain-specific effects and host-related factors to optimize the development of personalized probiotic strategies within precision nutrition frameworks for athletes.

## Supplemental Information

10.7717/peerj.20809/supp-1 Supplemental Information 1 Supplemental tables

10.7717/peerj.20809/supp-2 Supplemental Information 2 PRISMA checklist

## Additional Information and Declarations

### Data Availability

The following information was supplied regarding data availability: This is a systematic review/meta-analysis.
