# Supplementary material for: Effects of probiotic supplementation on immune and inflammatory markers in athletes: an umbrella review and re-analysis of published meta-analyses of randomised controlled trials
Source: PeerJ. 2026 Feb 26;14:e20809. doi: 10.7717/peerj.20809 (PMC12950187; doi:10.7717/peerj.20809)
Supplement: Supplemental Information 1 [file peerj-14-20809-s001.docx]

Supplementary Material

# SupplementaLiterature Search Strategies for Various Databases

**Table S1. Search strategies for PubMed database**

| **Search** | **Search entries for published Meta-analyses** | **Search entries for additional reanalysis** |
| --- | --- | --- |
| Date | December 1, 2025 | June 28, 2024 - December 15, 2025 |
| #1 | ("Athletes"[MeSH] OR Athlete OR "Professional Athletes" OR "Athlete, Professional" OR "Athletes, Professional" OR "Professional Athlete" OR "Elite Athletes" OR "Athlete, Elite" OR "Athletes, Elite" OR "Elite Athlete" OR "College Athletes" OR "Athlete, College" OR "Athletes, College" OR "College Athlete") | |
| #2 | ("Probiotics"[Mesh]) OR "Probiotic") | |
| #3 | ("Meta-Analysis"[MeSH] OR "systematic review" OR "meta analysis") | ("Randomized Controlled Trial"[Publication Type] OR "Randomized trial" OR "RCT" OR "Controlled trial") |
| #4 | (#1) AND (#2) AND (#3) | (#1) AND (#2) AND (#3) |

**Table S2. Search strategies for Web of Science database**

| **Search** | **Search entries for published Meta-analyses** | **Search entries for additional reanalysis** |
| --- | --- | --- |
| Date | December 1, 2025 | June 28, 2024 - December 15, 2025 |
| #1 | (TS=("Athletes" OR Athlete OR "Professional Athletes" OR "Athlete, Professional" OR "Athletes, Professional" OR "Professional Athlete" OR "Elite Athletes" OR "Athlete, Elite" OR "Athletes, Elite" OR "Elite Athlete" OR "College Athletes" OR "Athlete, College" OR "Athletes, College" OR "College Athlete")) | |
| #2 | (TS=("Probiotics" OR "Probiotic")) | |
| #3 | (TS=("Meta-Analysis" OR "systematic review" OR "meta analysis")) | (TS=("Randomized Controlled Trial" OR "Randomized trial" OR "RCT" OR "Controlled trial")) |
| #4 | #1 AND #2 AND #3 | #1 AND #2 AND #3 |

**Table S3. Search strategies for Cochrane Library**

| **Search** | **Search entries for published Meta-analyses** | **Search entries for additional reanalysis** |
| --- | --- | --- |
| Date | December 1, 2025 | January 2024 - Present |
| #1 | ("Athletes" OR "Athlete" OR "Professional Athletes" OR "Athlete, Professional" OR "Elite Athletes" OR "Athletes, Elite" OR "Elite Athlete" OR "College Athletes" OR "Athlete, College" OR "College Athlete") | |
| #2 | ("Probiotics" OR "Probiotic") | |
| #3 | ("Meta-Analysis" OR "systematic review" OR "meta analysis") | ("Randomized Controlled Trial" OR "Randomized trial" OR "RCT" OR "Controlled trial") |
| #4 | #1 AND #2 AND #3 | #1 AND #2 AND #3 |

**Table S4. Search strategies for Embase database**

| **Search** | **Search entries for published Meta-analyses** | **Search entries for additional reanalysis** |
| --- | --- | --- |
| Date | December 1, 2025 | June 28, 2024 - December 15, 2025 |
| #1 | ('athlete' OR 'athletes' OR 'professional athlete' OR 'professional athletes' OR 'elite athlete' OR 'elite athletes' OR 'college athlete' OR 'college athletes') | |
| #2 | ('probiotic' OR 'probiotics') | |
| #3 | ('meta-analysis' OR 'systematic review') | ('randomized controlled trial' OR 'randomized trial' OR 'RCT' OR 'controlled trial') |
| #4 | #1 AND #2 AND #3 | #1 AND #2 AND #3 |

**Table S5. Search strategies for Scopus database**

| **Search** | **Search entries for published Meta-analyses** | **Search entries for additional reanalysis** |
| --- | --- | --- |
| Date | December 1, 2025 | June 28, 2024 - December 15, 2025 |
| #1 | TITLE-ABS-KEY('athlete' OR 'athletes' OR 'professional athlete' OR 'professional athletes' OR 'elite athlete' OR 'elite athletes' OR 'college athlete' OR 'college athletes') | |
| #2 | TITLE-ABS-KEY('probiotic' OR 'probiotics') | |
| #3 | TITLE-ABS-KEY('meta-analysis' OR 'systematic review') | TITLE-ABS-KEY( 'randomized controlled trial' OR 'randomized trial' OR 'RCT' OR 'controlled trial') |
| #4 | #1 AND #2 AND #3 | #1 AND #2 AND #3 |

# Characteristics of the original literature materials in the meta-analysis.

# Table S6. Table of Athlete Characteristics in Original Studies Included in the Meta-Analysis.

| **Included study**  **(first author, year)** | **Original study**  **(first author, year)** | **Athlete type** | **Age (years)** | **Sex & sample size** |
| --- | --- | --- | --- | --- |
| Aparicio-Pascual  2025 | Axelrod 2019 | Endurance trained athletes | 18 – 45 | Sex not reported (n=7) |
|  | Batatinh 2020 | Marathon runners | 30 – 45 | 27 M |
|  | Enge 2022 | Endurance athletes | 18 – 50 | Sex not reported (n=126) |
|  | Gil 2016 | Non-heat acclimatized endurance athletes | 26 – 46 | 8 M |
|  | Huang 2019 | Triathletes | 20.65 ± 1.1 – 21.2 ± 0.75 | Sex not reported (n=37) |
|  | Jäger 2016 | Resistance-trained athletes | 25 ± 4 | 15 M |
|  | Lamprecht 2012 | Endurance trained athletes | 30 – 45 | 23 M |
|  | Lennon 2024] | Runners | 32.7 ± 8 | 16 M & F |
|  | Mazani 2018 | Athletes (sport not specified) | 18 – 25 | 27F |
|  | Mazur-Kurach 2022 | Elite road cyclists | 18 – 26 | 26M |
|  | Pugh 2019 | Runners | 35.45 ± 7.2 | 20 M & F |
|  | Pugh 2020 | Trained cyclists | 24 ± 1 | Sex not reported (n=7) |
|  | Shing 2014 | Trained runners | 27 ± 2 | 10M |
|  | Tavares-Silva 2021 | Marathon runners | 39.92 ± 3.47 | 14 M |
|  | Tavares-Silva 2024 | Marathon runners | 25 – 45 | 27 M |
|  | Vaiseberg 2019 | Amateur marathon runners | 39.5 ± 9.4 | 42 M |
|  | West 2011 | Cyclists and triathletes | 35.2 ± 10.3 – 36.05 ± 9.4 | 35F / 62M |
| Tavakoly  2021 | Cox 2014 | Triathlon, running | 39.46 ± 0.5 | 129 (M & F) |
|  | Donmez 2014 | Endurance | 33.66 ± 0.5 | 18 M |
|  | Gleeson 2011 | Running, cycling, swimming | 27 ± 5 | 54 M / 30 F |
|  | Gleeson 2012 | Running, cycling, swimming | 23.9 ± 0.5 | 28 M / 38 F |
|  | Gleeson 2016 | Running, cycling, swimming, team ball sports | 20.45 ± 0.2 | 156 M / 112 F |
|  | Ibrahim 2018 | Strength sports | 22 ± 0.5 | 41 M |
|  | Marinkovic 2016 | Endurance | 23.15 ± 0.1 | 36 M / 14 F |
|  | Pumpa 2019 | Rugby | 26.79 ± 0.5 | 19 M |
|  | Sawada 2019 | Endurance | 19.9 ± 0.5 | 49 M |
|  | Shing 2014 | Resistance training | 27 ± 0.5 | 10 M |
|  | Tiollier 2007 | Endurance | 21 | 47 M |
|  | Townsend 2018 | Baseball | 20.1 ± 1.5 | 25 M |
|  | West 2011 | Cycling, triathlon | 35.2 ± 10.3 | 29 M / 33 F |
| Guo  2022 | Batatinha 2020 | Marathon | 32 ± 14 | 27 M |
|  | Gleeson 2011 | Endurance | 35.96 ± 5.81 | 84 (M & F) |
|  | Pugh 2019 | Marathon | 34.8 ± 6.9 | 20 M / 4 F |
|  | Quero 2021 | Soccer | NR | 20 M / 4 F |
|  | Quero-Calero 2022 | Soccer | 20.66 ± 1.39 | 13 M |
|  | Schreiber 2021 | Cycling | 25.9 ± 4.6 | 13 M |
|  | Smarkusz-Zarzecka 2020 | Endurance | 39.35 ± 8.23 | 46 M / 20 F |
|  | Tavares-Silva 2021 | Marathon | NR | 14 M |
|  | West 2011 | Cycling, triathlon | 35.2 ± 10.3 | 29 M / 33 F |
| Nazari  2020 | Brennan 2018 | Endurance | 31 ± 0.5 | 5 M / 2 F |
|  | Cox 2010 | Triathlon | 27.3 ± 0.5 | 20 M |
|  | Gepner 2017 | Military training | 20 ± 0.5 | 26 M |
|  | Gleeson 2011 | Running, cycling | 27 ± 0.5 | 54 M / 30 F |
|  | Huang 2019 Study 1 | Triathlon | 20.65 ± 0.5 | 18 M |
|  | Huang 2019 Study 2 | Triathlon | 21.2 ± 0.5 | 16 M |
|  | Ibrahim 2018 | Resistance training | 22 ± 0.5 | 48 M |
|  | Jager 2016 | Resistance training | 25 ± 0.5 | 15 M |
|  | Lamprecht 2012 | Strength training | 37.9 ± 0.5 | 23 M |
|  | Marinkovic 2016 | Aerobic endurance | 23.15 ± 0.1 | 36 M / 14 F |
|  | Mazani 2018 | Resistance training | 18 ± 5 | 27 F |
|  | Pugh 2019 | Marathon | 35.4 ± 0.5 | 20 M / 4 F |
|  | Shing 2014 | Resistance training | 27 ± 0.5 | 10 M |
|  | Townsend 2018 | Baseball | 20.1 ± 1.5 | 25 M |
| Łagowska & Bajerska  2021 | Gill 2016 | Endurance | 26 ± 6 | 8 M / 8 F |
|  | Gleeson 2011 | Running, cycling, swimming | 32 ± 14 | 32 M / 26 F |
|  | Gleeson 2012 | Running, cycling, swimming | 25 ± 5 | 27 M / 27 F |
|  | Gleeson 2016 | Running, cycling, swimming | 20.3 ± 0.2 | 126 M / 117 F |
|  | Haywood 2014 | Rugby | 24.7 ± 3.6 | 30 M / 30 F |
|  | Huang 2019 Study 1 | Triathlon | 19 ± 4 | 9 M / 9 F |
|  | Huang 2019 Study 2 | Triathlon | 19 ± 1 | 8 M / 8 F |
|  | Kekkonen 2007 | Marathon | 40 (22–69) | 61 M / 58 F |
|  | Khani 2018 | Sprinting | 21 ± 3 | 53 M / 3 F |
|  | Michalickova 2016 | Multi-sport (swimming, cycling, mountaineering, etc.) | 23.5 ± 2.7 | 20 M / 19 F |
|  | Pugh 2019 | Marathon | 34.8 ± 6.9 | 12 M / 12 F |
|  | Pumpa 2019 | Rugby | 27.0 ± 3.2 | 9 M / 10 F |
|  | Townsend 2018 | Baseball | 20.1 ± 1.5 | 13 M / 12 F |
|  | West 2011 | Cycling, triathlon | 35.2 ± 10.3 | 29 M / 33 F |
| Abbreviations: M, male; F, female; NR, not reported. | | | | |

# Supplement the GRADE Evidence Quality Overview

**Table S7. GRADE Evidence Profile: Impact of Probiotic Supplementation on Immune and Inflammatory Biomarkers in Athletes**

| **Reference** | **Outcomes** | **Risk of biases** | **Inconsistency** | **Indirectness** | **Imprecision** | **Other bias** | **GRADE level** |
| --- | --- | --- | --- | --- | --- | --- | --- |
| Aparicio-Pascua, 2025 | TNF-α | Serious ^a^ | Very serious ^b^ | Not serious | Very serious ^d^ | No Publication bias | ⚪⚪⚪⚫  Very low |
|  | IFN-y | Serious ^a^ | Very serious ^b^ | Not serious | Very serious ^d^ | No Publication bias | ⚪⚪⚪⚫  Very low |
|  | IL-6 | Serious ^a^ | Not serious | Not serious | Serious ^c^ | Publication bias suspecte ^d^ | ⚫⚪⚫⚫  Moderate |
|  | IL-8 | Serious ^a^ | Serious ^b^ | Not serious | Serious ^c^ | No Publication bias | ⚫⚫⚪⚫  Low |
|  | IL-10 | Serious ^a^ | Not serious | Not serious | Not serious | No Publication biasz | ⚫⚫⚫⚪ High |
| Guo,2022 | TNF-α | Very serious ^a^ | Very serious ^b^ | Not serious | Very serious ^d^ | No Publication bias | ⚪⚪⚫⚪  Very low |
|  | IFN-y | Not serious | Very serious ^b^ | Not serious | Very serious ^d^ | No Publication bias | ⚫⚪⚫⚪  Low |
|  | IL-6 | Very serious ^a^ | Not serious | Not serious | Very serious ^d^ | No Publication bias | ⚪⚫⚫⚪  Low |
|  | IL-8 | Very serious ^a^ | Very serious ^b^ | Not serious | Very serious ^d^ | No Publication bias | ⚪⚪⚫⚪  Very low |
|  | IL-10 | Very serious ^a^ | Not serious | Not serious | Not serious | No Publication bias | ⚪⚫⚫⚫  Moderate |
|  | IgA | Not serious | Not serious | Not serious | Very serious ^d^ | No Publication bias | ⚫⚫⚫⚪  Moderate |
| Maryam,2020 | TNF-α | Very serious ^a^ | Not serious | Very serious ^c^ | Very serious ^d^ | No Publication bias | ⚫⚪⚫⚫  Very low |
|  | IFN-y | Very serious ^a^ | Very serious ^b^ | Not serious | Very serious ^d^ | No Publication bias | ⚫⚫⚪⚫  Very low |
|  | IL-6 | Very serious ^a^ | Not serious | Not serious | Not serious | No Publication bias | ⚪⚫⚫⚫  Moderate |
|  | IL-8 | Very serious ^a^ | Not serious | Very serious ^c^ | Not serious | No Publication bias | ⚪⚫⚪⚫  Low |
|  | IL-10 | Very serious ^a^ | Not serious | Very serious ^c^ | Not serious | No Publication bias | ⚪⚫⚪⚫  Low |
| Karolina,2021 | TNF-α | Very serious ^a^ | Very serious ^b^ | Not serious | Very serious ^d^ | No Publication bias | ⚪⚪⚫⚪  Very low |
|  | IgA | Very serious ^a^ | Not serious | Not serious | Very serious ^d^ | No Publication bias | ⚪⚫⚫⚪  Low |
|  | IL-6 | Very serious ^a^ | Very serious ^b^ | Not serious | Very serious ^d^ | No Publication bias | ⚪⚪⚫⚪  Very low |
|  | IL-10 | Very serious ^a^ | Very serious ^b^ | Not serious | Very serious ^d^ | No Publication bias | ⚪⚪⚫⚪  Very low |
| Rahele,2021 | IgA | Very serious ^a^ | Not serious | Not serious | Not serious | No Publication bias | ⚪⚫⚫⚫  Moderate |
| a: Heterogeneity is high (I^2^>50%);b: More than half of the trials used less than sample size of 50 per group, with wide confidence intervals;c: More than half of the trials have wide confidence intervals;d: Publication bias was not assessed or was likely to be high. | | | | | | | |

# Corrected covered area

**Table S8. Corrected Covered Area (CCA) Analysis of TNF-α-Related Studies**

| **Primary Studies** | | **Systematic Reviews (Mandatory)** | | | | |
| --- | --- | --- | --- | --- | --- | --- |
| **Study ID (Mandatory)** | **Reference (Optional)** | **Guo 2022** | **Maryam 2020** | **Karolina 2021** | **Aparicio‑Pascual 2025** | |
| Batatinha 2020 |  | 1 |  |  | 2 | |
| Gleeson 2011 |  | 1 |  |  |  | |
| Quero-Calero 2022 |  | 1 |  |  |  | |
| Schreiber 2021 |  | 1 |  |  |  | |
| Smarkusz-Zarzecka 2020 |  | 1 |  |  | 2 | |
| Tavares-Silva 2021 |  | 1 |  |  | 2 | |
| West 2011 |  | 1 |  | 2 | 3 | |
| Huang 2019 |  |  | 1 | 2 | 3 | |
| Mazani 2018 |  |  | 1 |  | 2 | |
| Townsend 2018 |  |  | 1 | 2 |  | |
| Lamprecht 2012 |  |  | 1 |  | 2 | |
| Vasiberg 2019 |  |  |  |  | 1 | |
| Mazur-Kurach 2022 |  |  |  |  | 1 | |
| Gill 2016 |  |  |  |  | 1 | |
| Tavares-Silva 2024 |  |  |  |  | 1 | |
| Lennon 2024 |  |  |  |  | 1 | |
| Engel 2022 |  |  |  |  | 1 | |
| Shing 2014 |  |  |  |  | 1 | |
| **Corrected covered area** | | **(N-r)/(rc-r)** | | | | **18.52%** |

**Table S9. Corrected Covered Area (CCA) Analysis of LgA-Related Studies**

| **Primary Studies** | | **Systematic Reviews (Mandatory)** | | |
| --- | --- | --- | --- | --- |
| **Study ID (Mandatory)** | **Reference (Optional)** | **Guo 2022** | **Karolina 2021** | **Rahele 2021** |
| Quero 2021 |  | 1 |  |  |
| Tavares-Silva 2021 |  | 1 |  |  |
| Gill 2016 |  |  | 1 |  |
| Ibrahim 2018 |  |  |  | 1 |
| Gleeson 2011 |  |  |  | 1 |
| Pumpa 2019 |  |  | 1 | 2 |
| Gleeson 2012 |  |  | 1 | 2 |
| Khani 2018 |  |  | 1 |  |
| **Corrected covered area** | | **(N-r)/(rc-r)** | | **12.50%** |

**Table S10. Corrected Covered Area (CCA) Analysis of IFN-y-Related Studies**

| **Primary Studies** | | **Systematic Reviews (Mandatory)** | | |
| --- | --- | --- | --- | --- |
| **Study ID (Mandatory)** | **Reference (Optional)** | **Guo 2022** | **Maryam 2020** | **Aparicio‑Pascual 2025** |
| West 2011 |  | 1 |  | 2 |
| Batatinha 2020 |  | 1 |  | 2 |
| Marinkovic 2016 |  |  | 1 |  |
| Huang 2019 |  |  | 1 |  |
| Cox 2010 |  |  | 1 |  |
| Gill 2016 |  |  |  | 1 |
| Engel 2022 |  |  |  | 1 |
| **Corrected covered area** | | **(N-r)/(rc-r)** | | **0%** |

**Table S11. Corrected Covered Area (CCA) Analysis of IL-6-Related Studies**

| **Primary Studies** | | **Systematic Reviews(Mandatory)** | | | |
| --- | --- | --- | --- | --- | --- |
| **Study ID (Mandatory)** | **Reference (Optional)** | **Guo 2022** | **Maryam 2020** | **Karolina 2021** | **Aparicio‑Pascual 2025** |
| Batatinha 2020 |  | 1 |  |  | 2 |
| Pugh 2019 |  | 1 | 2 | 3 | 4 |
| Quero-Calero 2022 |  | 1 |  |  |  |
| Schreiber 2021 |  | 1 |  |  |  |
| West 2011 |  | 1 |  | 2 | 3 |
| Jager 2016 |  |  | 1 |  |  |
| Huang 2019 |  |  | 1 | 2 | 3 |
| Mazani 2018 |  |  | 1 |  |  |
| Lamprecht 2012 |  |  | 1 |  |  |
| Brennan 2018 |  |  | 1 |  |  |
| Ibrahim 2018 |  |  | 1 |  |  |
| Shing 2014 |  |  | 1 |  |  |
| Axelrod 2019 |  |  |  |  | 1 |
| Tavares-Silva 2024 |  |  |  |  | 1 |
| Gill 2016 |  |  |  |  | 1 |
| Mazur-Kurach 2022 |  |  |  |  | 1 |
| Engel 2022 |  |  |  |  | 1 |
| Lennon 2024 |  |  |  |  | 1 |
| **Corrected covered area** | | **(N-r)/(rc-r)** | | **22.22%** | |

**Table S12. Corrected Covered Area (CCA) Analysis of IL-8-Related Studies**

| **Primary Studies** | | **Systematic Reviews (Mandatory)** | | |
| --- | --- | --- | --- | --- |
| **Study ID (Mandatory)** | **Reference (Optional)** | **Guo 2022** | **Maryam 2020** | **Aparicio‑Pascual 2025** |
| Batatinha 2020 |  | 1 |  | 1 |
| Pugh 2019 |  | 1 | 2 | 3 |
| Quero-Calero 2022 |  | 1 |  |  |
| West 2011 |  | 1 |  | 2 |
| Huang 2019 |  |  | 1 | 2 |
| Mazur-Kurach 2022 |  |  |  | 1 |
| Pugh 2020 |  |  |  | 1 |
| Tavares-Silva 2024 |  |  |  | 1 |
| Engel 2022 |  |  |  | 1 |
| Gill 2016 |  |  |  | 1 |
| Lennon 2024 |  |  |  | 1 |
| **Corrected covered area** | | **(N-r)/(rc-r)** | | **22.73%** |

**Table S13. Corrected Covered Area (CCA) Analysis of IL-10-Related Studies**

| **Primary Studies** | | **Systematic Reviews (Mandatory)** | | | |
| --- | --- | --- | --- | --- | --- |
| **Study ID (Mandatory)** | **Reference (Optional)** | **Guo 2022** | **Maryam 2020** | **Karolina 2021** | **Aparicio‑Pascual 2025** |
| Batatinha 2020 |  | 1 |  |  | 1 |
| Pugh 2019 |  | 1 | 2 | 3 | 4 |
| Quero-Calero 2022 |  | 1 |  |  |  |
| Tavares-Silva 2021 |  | 1 |  |  | 2 |
| West 2011 |  | 1 |  | 2 | 3 |
| Marinkovic 2016 |  |  | 1 |  |  |
| Huang 2019 |  |  | 1 |  | 2 |
| Townsend 2018 |  |  | 1 | 2 |  |
| Ibrahim 2018 |  |  | 1 |  |  |
| Michalickova 2016 |  |  |  | 1 |  |
| Gill 2016 |  |  |  |  | 1 |
| Pugh 2020 |  |  |  |  | 1 |
| Lennon 2024 |  |  |  |  | 1 |
| Mazur-Kurach 2022 |  |  |  |  | 1 |
| Vasiverg 2019 |  |  |  |  | 1 |
| Tavares-Silva 2024 |  |  |  |  | 1 |
| Engel 2022 |  |  |  |  | 1 |
| Shing 2014 |  |  |  |  | 1 |
| **Corrected covered area** | | **(N-r)/(rc-r)** | | | **16.67%** |

**References**

**Batatinha, H., TavaresSilva, E., Leite, G. S. F., Resende, A. S., Albuquerque, J. A. T., Arslanian, C., Fock, R. A., Lancha, A. H., Lira, F. S., Krüger, K., ThomatieliSantos, R., & RosaNeto, J. C. 2020.** Probiotic supplementation in marathonists and its impact on lymphocyte population and function after a marathon: a randomized placebo-controlled double-blind study. *Scientific Reports*, *10*(1): 18777-18777. <https://doi.org/10.1038/s41598-020-75464-0>

**Gleeson, M., C, B. N., Marta, O., & Pedro, T. 2011.** Daily probiotic's (Lactobacillus casei Shirota) reduction of infection incidence in athletes. *International journal of sport nutrition and exercise metabolism*, *21*(1): 55-64.

**Quero-Calero, C. D., AbellánAynés, O., Manonelles, P., & Ortega, E. 2022.** The Consumption of a Synbiotic Does Not Affect the Immune, Inflammatory, and Sympathovagal Parameters in Athletes and Sedentary Individuals: A Triple-Blinded, Randomized, Place-bo-Controlled Pilot Study. *Int J Environ Res Public Health*, *19*(6): 3421-3421. <https://doi.org/10.3390/ijerph19063421>

**Schreiber, C., Tamir, S., Golan, R., Weinstein, A., & Weinstein, Y. 2021.** The effect of probiotic supplementation on performance, inflammatory markers and gastro-intestinal symptoms in elite road cyclists. *Journal of the International Society of Sports Nutrition*, *18*(1): 36-36. <https://doi.org/10.1186/s12970-021-00432-6>

**Smarkusz-Zarzecka, J., Ostrowska, L., Leszczyńska, J., Orywal, K., Cwalina, U., & Pogodziński, D. 2020.** Analysis of the Impact of a Multi-Strain Probiotic on Body Composition and Cardiorespiratory Fitness in Long-Distance Runners. *Nutrients*, *12*(12). <https://doi.org/10.3390/nu12123758>

**Tavares-Silva, E., Caris, A. V., Santos, S. A., Ravacci, G. R., & ThomatieliSantos, R. V. 2021.** Effect of Multi-Strain Probiotic Supplementation on URTI Symptoms and Cytokine Production by Monocytes after a Marathon Race: A Randomized, Double-Blind, Placebo Study. *Nutrients*, *13*(5): 1478-1478. <https://doi.org/10.3390/nu13051478>

**West, N. P., Pyne, D. B., Cripps, A. W., Hopkins, W. G., Eskesen, D. C., Jairath, A., Christophersen, C. T., Conlon, M. A., & Fricker, P. A. 2011.** Lactobacillus fermentum (PCC®) supplementation and gastrointestinal and respiratory-tract illness symptoms: a randomised control trial in athletes. *Nutr J*, *10*: 30. <https://doi.org/10.1186/1475-2891-10-30>

**Huang, W. C., Lee, M. C., Lee, C. C., Ng, K. S., Hsu, Y. J., Tsai, T. Y., Young, S. L., Lin, J. S., & Huang, C. C. 2019.** Effect of Lactobacillus plantarum TWK10 on Exercise Physiological Adaptation, Performance, and Body Composition in Healthy Humans. *Nutrients*, *11*(11). <https://doi.org/10.3390/nu11112836>

**Mazani, M., Nemati, A., Amani, M., Haedari, K., Mogadam, R. A., & Baghi, A. N. 2018.** The effect of probiotic yoghurt consumption on oxidative stress and inflammatory factors in young females after exhaustive exercise. *J Pak Med Assoc*, *68*(12): 1748-1754.

**Townsend, J. R., Bender, D., Vantrease, W. C., Sapp, P. A., Toy, A. M., Woods, C. A., & Johnson, K. D. 2018.** Effects of Probiotic (Bacillus subtilis DE111) Supplementation on Immune Function, Hormonal Status, and Physical Performance in Division I Baseball Players. *Sports*, *6*(3): 70-70. <https://doi.org/10.3390/sports6030070>

**Lamprecht, M., Bogner, S., Schippinger, G., Steinbauer, K., Fankhauser, F., Hallstroem, S., Schuetz, B., & Greilberger, J. F. 2012.** Probiotic supplementation affects markers of intestinal barrier, oxidation, and inflammation in trained men; a randomized, double-blinded, placebo-controlled trial. *Journal of the International Society of Sports Nutrition*, *9*(1): 45. <https://doi.org/10.1186/1550-2783-9-45>

**Vaisberg, M., Paixão, V., Almeida, E. B., Santos, J. M. B., Foster, R., Rossi, M., Pithon-Curi, T. C., Gorjão, R., Momesso, C. M., Andrade, M. S., Araujo, J. R., Garcia, M. C., Cohen, M., Perez, E. C., Santos-Dias, A., Vieira, R. P., & Bachi, A. L. L. 2019.** Daily Intake of Fermented Milk Containing Lactobacillus casei Shirota (Lcs) Modulates Systemic and Upper Airways Immune/Inflammatory Responses in Marathon Runners. *Nutrients*, *11*(7): 1678-1678. <https://doi.org/10.3390/nu11071678>

**Mazur-Kurach, P., Frączek, B., & Klimek Andrzej, T. 2022.** Does Multi-Strain Probiotic Supplementation Impact the Effort Capacity of Competitive Road Cyclists? *Int J Environ Res Public Health*, *19*(19): 12205-12205. <https://doi.org/10.3390/ijerph191912205>

**Gill, S. K., Teixeira, A. M., Rosado, F., Cox, M., & Costa, R. J. 2016.** High-Dose Probiotic Supplementation Containing Lactobacillus casei for 7 Days Does Not Enhance Salivary Antimicrobial Protein Responses to Exertional Heat Stress Compared With Placebo. *Int J Sport Nutr Exerc Metab*, *26*(2): 150-160. <https://doi.org/10.1123/ijsnem.2015-0171>

**Tavares-Silva, E., Leite, G. S., Batatinha, H. A., Resende, A. d. S., Lemos, V. d. A., Marques, C. G., Junior, A. H. L., Neto, J. C. R., & Santos, R. V. T. 2024.** Thirty days of double-strain probiotic supplementation increases monocyte phagocytosis in marathon runners. *The British journal of nutrition*, *132*(3): 21-24. <https://doi.org/10.1017/s0007114524001259>

**Lennon, S., Lackie, T., Miltko, A., Kearns, Z. C., Paquette, M., Bloomer, R. J., Wang, A., & Merwe, M. v. d. 2024.** Safety and Efficacy of a probiotic cocktail containing P. acidilatici and L. plantarum for gastrointestinal discomfort in endurance runners: Randomized Double-Blinded Crossover Clinical Trial. *Applied physiology, nutrition, and metabolism = Physiologie appliquee, nutrition et metabolisme*. <https://doi.org/10.1139/apnm-2023-0449>

**Engel, S., Mortensen, B., Wellejus, A., VeraJimenez, N., Struve, C., Brummer, R. J., Damholt, A., Woods, T., & Shanahan, F. 2022.** Safety of Bifidobacterium breve, Bif195, employing a human exercise-induced intestinal permeability model: a randomised, double-blinded, placebo-controlled, parallel group trial. *Beneficial microbes*, *13*(3): 11-10. <https://doi.org/10.3920/bm2021.0173>

**Shing, C. M., Peake, J. M., Lim, C. L., Briskey, D., Walsh, N. P., Fortes, M. B., Ahuja, K. D., & Vitetta, L. 2014.** Effects of probiotics supplementation on gastrointestinal permeability, inflammation and exercise performance in the heat. *Eur J Appl Physiol*, *114*(1): 93-103. <https://doi.org/10.1007/s00421-013-2748-y>

**Quero, C. D., Manonelles, P., Fernández, M., Abellán-Aynés, O., López-Plaza, D., Andreu-Caravaca, L., Hinchado, M. D., Gálvez, I., & Ortega, E. 2021.** Differential Health Effects on Inflammatory, Immunological and Stress Parameters in Professional Soccer Players and Sedentary Individuals after Consuming a Synbiotic. A Triple-Blinded, Randomized, Placebo-Controlled Pilot Study. *Nutrients*, *13*(4). <https://doi.org/10.3390/nu13041321>

**Ibrahim, N. S., Ooi, F. K., Chen, C. K., & Muhamad, A. S. 2018.** Effects of probiotics supplementation and circuit training on immune responses among sedentary young males. *J Sports Med Phys Fitness*, *58*(7-8): 1102-1109. <https://doi.org/10.23736/s0022-4707.17.07742-8>

**Pumpa, K. L., McKune, A. J., & Harnett, J. 2019.** A novel role of probiotics in improving host defence of elite rugby union athlete: A double blind randomised controlled trial. *J Sci Med Sport*, *22*(8): 876-881. <https://doi.org/10.1016/j.jsams.2019.03.013>

**Gleeson, M., Bishop, N. C., Oliveira, M., McCauley, T., Tauler, P., & Lawrence, C. 2012.** Effects of a Lactobacillus salivarius probiotic intervention on infection, cold symptom duration and severity, and mucosal immunity in endurance athletes. *Int J Sport Nutr Exerc Metab*, *22*(4): 235-242. <https://doi.org/10.1123/ijsnem.22.4.235>

Hossein Khani, A., Mousavi Jazayeri, S. M., Ebarhimi, E., & Farhadi, A. (2019). The Bifidobacterim bifidum (BIB2) Probiotic Increased Immune System Factors in Men Sprint Athletes. In M. Chávarri Hueda (Ed.), *Nutraceuticals - Past, Present and Future*. IntechOpen. <https://doi.org/10.5772/intechopen.84222>

**Michalickova, D., Minic, R., Dikic, N., Andjelkovic, M., Kostic-Vucicevic, M., Stojmenovic, T., Nikolic, I., & Djordjevic, B. 2016.** Lactobacillus helveticus Lafti L10 supplementation reduces respiratory infection duration in a cohort of elite athletes: a randomized, double-blind, placebo-controlled trial. *Appl Physiol Nutr Metab*, *41*(7): 782-789. <https://doi.org/10.1139/apnm-2015-0541>

**Cox, A. J., Pyne, D. B., Saunders, P. U., & Fricker, P. A. 2010.** Oral administration of the probiotic Lactobacillus fermentum VRI-003 and mucosal immunity in endurance athletes. *Br J Sports Med*, *44*(4): 222-226. <https://doi.org/10.1136/bjsm.2007.044628>

**Pugh, J. N., Sparks, A. S., Doran, D. A., Fleming, S. C., Langan-Evans, C., Kirk, B., Fearn, R., Morton, J. P., & Close, G. L. 2019.** Four weeks of probiotic supplementation reduces GI symptoms during a marathon race. *Eur J Appl Physiol*, *119*(7): 1491-1501. <https://doi.org/10.1007/s00421-019-04136-3>

**Jäger, R., Purpura, M., Stone, J. D., Turner, S. M., Anzalone, A. J., Eimerbrink, M. J., Pane, M., Amoruso, A., Rowlands, D. S., & Oliver, J. M. 2016.** Probiotic Streptococcus thermophilus FP4 and Bifidobacterium breve BR03 Supplementation Attenuates Performance and Range-of-Motion Decrements Following Muscle Damaging Exercise. *Nutrients*, *8*(10). <https://doi.org/10.3390/nu8100642>

**Brennan, C. J., Axelrod, C. L., Paul, D., Hull, M., & Kirwan, J. P. 2018.** Effects Of A Novel Probiotic On Exercise-Induced Gut Permeability and Microbiota in Endurance Athletes: 3372 Board #. *Medicine & Science in Sports & Exercise*, *50*(5S): 840. <https://doi.org/10.1249/01.mss.0000538764.55259.9c>

**Lennon, S., Lackie, T., Miltko, A., Kearns, Z. C., Paquette, M. R., Bloomer, R. J., Wang, A., & van der Merwe, M. 2024.** Safety and efficacy of a probiotic cocktail containing P. acidilactici and L. plantarum for gastrointestinal discomfort in endurance runners: randomized double-blinded crossover clinical trial. *Appl Physiol Nutr Metab*, *49*(7): 890-903. <https://doi.org/10.1139/apnm-2023-0449>

**Pugh, J. N., Wagenmakers, A. J. M., Doran, D. A., Fleming, S. C., Fielding, B. A., Morton, J. P., & Close, G. L. 2020.** Probiotic supplementation increases carbohydrate metabolism in trained male cyclists: a randomized, double-blind, placebo-controlled crossover trial. *American Journal of Physiology-Endocrinology and Metabolism*, *318*(4): E504-E513. <https://doi.org/10.1152/ajpendo.00452.2019>

**Townsend, J. R., Bender, D., Vantrease, W. C., Sapp, P. A., Toy, A. M., Woods, C. A., & Johnson, K. D. 2018.** Effects of Probiotic (Bacillus subtilis DE111) Supplementation on Immune Function, Hormonal Status, and Physical Performance in Division I Baseball Players. *Sports (Basel)*, *6*(3). <https://doi.org/10.3390/sports6030070>

**Michalickova, D. M., Kostic-Vucicevic, M. M., Vukasinovic-Vesic, M. D., Stojmenovic, T. B., Dikic, N. V., Andjelkovic, M. S., Djordjevic, B. I., Tanaskovic, B. P., & Minic, R. D. 2017.** Lactobacillus helveticus Lafti L10 Supplementation Modulates Mucosal and Humoral Immunity in Elite Athletes: A Randomized, Double-Blind, Placebo-Controlled Trial. *J Strength Cond Res*, *31*(1): 62-70. <https://doi.org/10.1519/jsc.0000000000001456>
